# Carotid artery wave intensity in mid- to late-life predicts cognitive decline: the Whitehall II study

**DOI:** 10.1093/eurheartj/ehz189

**Published:** 2019-04-08

**Authors:** Scott T Chiesa, Stefano Masi, Martin J Shipley, Elizabeth A Ellins, Alan G Fraser, Alun D Hughes, Riyaz S Patel, Ashraf W Khir, Julian P Halcox, Archana Singh-Manoux, Mika Kivimaki, David S Celermajer, John E Deanfield

**Affiliations:** 1 National Centre for Cardiovascular Preventions and Outcomes, UCL Institute of Cardiovascular Science, 1 St. Martin’s Le Grand, London, UK; 2 Department of Clinical and Experimental Medicine, Universitá di Pisa, Building 8, S. Chiara Hospital, Via Roma 67, Pisa, Italy; 3 Department of Epidemiology and Public Health, UCL, 1-19 Torrington Place, London, UK; 4 Institute of Life Science, Swansea University Medical School, Swansea University, Singleton Park, Swansea, UK; 5 School of Medicine, Heath Park, Cardiff, UK; 6 Department of Cardiology, University Hospital of Wales, Heath Park, Cardiff, UK; 7 Department of Population Science and Experimental Medicine, UCL Institute of Cardiovascular Science, 69-75 Chenies Mews, London, UK; 8 Medical Research Council Unit for Lifelong Health and Ageing at UCL, 33 Bedford Place, London, UK; 9 Department of Cardiology, Bart’s Heart Centre, St Bartholomew’s Hospital, W Smithfield, London, UK; 10 Biomedical Engineering Research Theme, Brunel University London, Kingston Lane, Uxbridge, UK; 11 Inserm U1153, Epidemiology of Ageing and Neurodegenerative Diseases, Faculty of Medicine, University of Paris, 10 Avenue de Verdun, Paris, France; 12 Heart Research Institute, Eliza Street, Newtown, NSW, Australia

**Keywords:** Wave intensity, Cardiovascular risk factors, Cognitive decline, Whitehall II study

## Abstract

**Aims:**

Excessive arterial pulsatility may contribute to cognitive decline and risk of dementia via damage to the fragile cerebral microcirculation. We hypothesized that the intensity of downstream-travelling pulsatile waves measured by wave intensity analysis in the common carotid artery during mid- to late-life would be associated with subsequent cognitive decline.

**Methods and results:**

Duplex Doppler ultrasound was used to calculate peak forward-travelling compression wave intensity (FCWI) within the common carotid artery in 3191 individuals [mean ± standard deviation (SD), age = 61 ± 6 years; 75% male] assessed as part of the Whitehall II study in 2003–05. Serial measures of cognitive function were taken between 2002–04 and 2015–16. The relationship between FCWI and cognitive decline was adjusted for sociodemographic variables, genetic and health-related risk factors, and health behaviours. Mean (SD) 10-year change in standardized global cognitive score was -0.39 (0.18). Higher FCWI at baseline was associated with accelerated cognitive decline during follow-up [difference in 10-year change of global cognitive score per 1 SD higher FCWI = −0.02 (95% confidence interval −0.04 to −0.00); *P* = 0.03]. This association was largely driven by cognitive changes in individuals with the highest FCWI [Q4 vs. Q1–Q3 = −0.05 (−0.09 to −0.01), *P* = 0.01], equivalent to an age effect of 1.9 years. Compared to other participants, this group was ∼50% more likely to exhibit cognitive decline (defined as the top 15% most rapid reductions in cognitive function during follow-up) even after adjustments for multiple potential confounding factors [odds ratio 1.49 (1.17–1.88)].

**Conclusion:**

Elevated carotid artery wave intensity in mid- to late-life predicts faster cognitive decline in long-term follow-up independent of other cardiovascular risk factors.

## Introduction

Cognitive impairment and dementia are major contributing factors to morbidity and mortality in the elderly, but the causes and risk factors responsible for their development are still poorly understood. With the worldwide incidence of dementia projected to triple within the next 30 years,^1^ the identification of mechanistic pathways responsible for its progression is vital for preventive strategies aiming to reduce disease prevalence in an ageing population.

Recent cross-sectional^2–5^ and prospective^6–8^ studies suggest a potential link between arterial phenotypes in mid-life and the development of adverse structural brain changes, accelerated cognitive decline, and increased risk of dementia.^7^^,^^9–12^ This relationship may become more pronounced from middle-age, when a combination of vascular ageing and long-term exposure to cardiovascular disease (CVD) risk factors interact to augment the intensity of arterial pulsatility via a disproportionate stiffening of the aorta and the subsequent transmission of excess wave energy into the major arteries supplying the brain.^13^^,^^14^ While this association is supported by studies using surrogate markers such as aortic pulse-wave velocity and carotid pulse pressure (PP),^2^^,^^6^^,^^7^^,^^11^ a direct relationship between the likely mechanism responsible for downstream small vessel damage—an increase in the intensity of pulsatile waves travelling through the carotid arteries towards the brain—and future cognitive decline has never been examined.

We, therefore, investigated this relationship in a large cohort of adults recruited to the Whitehall II study; an ongoing longitudinal study of >3000 participants with novel carotid wave intensity measures performed in mid- to-late-life and in whom serial measures of cognitive function were available over 11–14 years of follow-up. We hypothesized that the peak intensity of the carotid artery forward-travelling compression wave in mid- to late-life would be associated with subsequent cognitive decline, and in particular that those with the highest forward compression wave intensity (FCWI) would exhibit the most pronounced decline in cognitive function, independent of other well-established cardiovascular risk factors.

## Methods

### Study population

**Figure 1:**
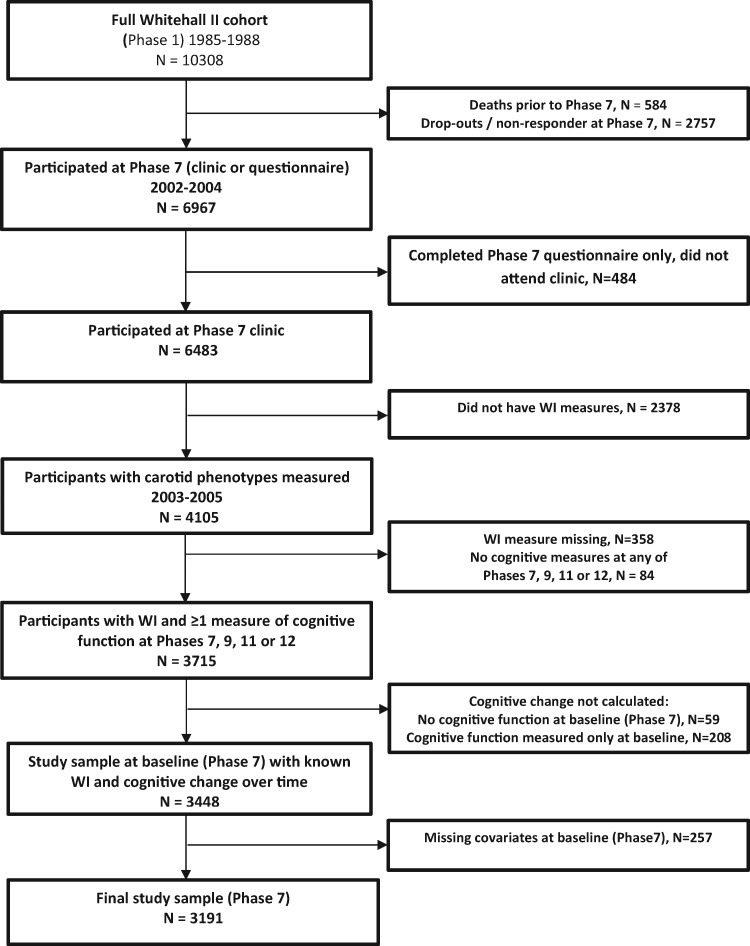
A cohort flowchart.

The Whitehall II Study is an ongoing cohort study of persons originally employed by the British Civil Service, full details of which have been reported previously.^15^ A total of 10 308 persons aged 33–55 years (67% male) were recruited to the study between 1985 and 1988. All participants responded to a comprehensive questionnaire and underwent a uniform, structured clinical evaluation, consisting of measures of anthropometry, cardiovascular and metabolic risk factors, and disease. Since baseline, follow-up examinations—which since 1997 (Phase 5) have additionally included tests of cognitive function—have taken place approximately every 5 years, with the most recent visit completed in 2015–16. In addition to official 5-year clinic visits, participants active in the study during the Phase 7 clinical examination (2002–04) were invited for a vascular clinic visit in 2003–05, in which wave intensity was measured in the carotid artery using duplex Doppler ultrasound. In the current analyses, we included only individuals with baseline measures of carotid wave intensity in 2003–05 and longitudinal measures of cognitive function assessed between 2002–04 and 2015–16 (*Figure 1*). All participants provided written consent prior to commencing the study. All protocols were approved by the University College London ethics committee and conformed to the Declaration of Helsinki.

**Take home figure:**
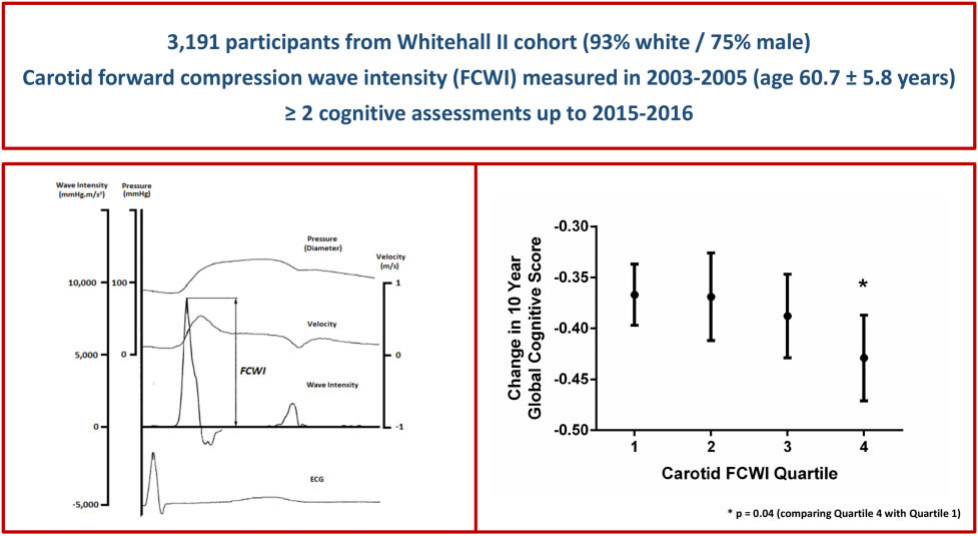
Elevated carotid artery wave intensity in mid- to late-life predicts faster cognitive decline in long-term follow-up independent of other cardiovascular risk factors.

### Wave intensity analysis

Carotid artery wave intensity (WI) was measured in 4105 participants (age 51–73 years, 75% male) attending a specialist vascular clinic conducted ∼1 year after the Phase 7 clinic. All participants who attended Phase 7 were invited to return to the WI clinic, and a comparison of those who accepted or refused is provided in Supplementary material online, *Table S1*. Intra-session intra- and inter-observer reproducibility analyses were conducted in a subset of participants (*n* = 39) and were in agreement with values reported in the literature (coefficient of variation = 10% and 19% for intra- and inter-observer repeat scans, respectively).

**Figure 2:**
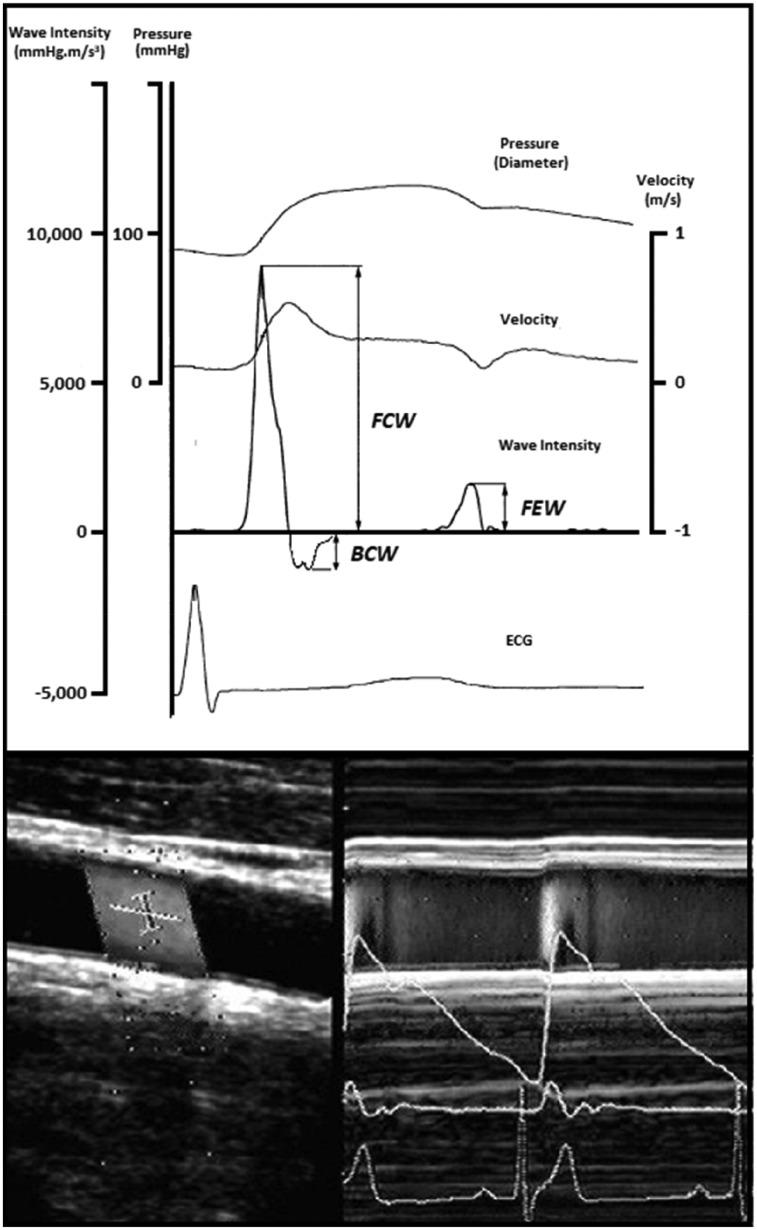
Representative image of a carotid wave intensity measurement performed using Doppler ultrasound. Representative trace showing output of carotid wave intensity analysis calculated from simultaneous non-invasive ultrasound measures of blood velocity (bottom left) and vessel diameter (bottom right). BCW, backwards compression wave; ECG, electrocardiogram; FCW, forward compression wave; FEW, forward expansion wave.

Full details of the WI technique and its clinical applicability have been published elsewhere.^16^ In brief, WI analysis provides a non-invasive measure of the net energy flux carried by forward- and backwards-travelling waves within the arterial system. During each cardiac cycle, left ventricular contraction results in the generation of a large forward-travelling compression wave in early systole, when blood velocity and pressure increase in tandem. This is often followed shortly afterwards by a reflected backwards-travelling compression wave in mid-systole—slowing velocity while continuing to increase pressure—and finally a forward-travelling expansion wave generated by the onset of left ventricular relaxation at the end of systole (*Figure 2*). For the purposes of this study, the peak intensity of the initial compression wave travelling towards the brain during early systole—i.e. FCWI—was our main focus. All measurements of FCWI were made using a colour Doppler ultrasound system (SSD-500, Aloka, Tokyo, Japan) fitted with a 7.5 MHz linear array probe. Independently, steerable ultrasound beams were used to measure simultaneous changes in arterial diameter and blood velocity in the common carotid artery at a site located ∼2 cm proximal to the carotid bulb (*Figure 2*). Automated software (Aloka, Tokyo, Japan) was then used to convert changes in diameter to estimates of pressure within the vessel (calibrated using brachial blood pressure). Time-normalized FCWI was calculated as the product of the time derivatives of the concurrent blood pressure (*dP*/*dt*) and velocity (*dU*/*dt*) waveforms.^17^ Measures were taken in both left and right carotid arteries, and the mean was used for analyses.

### Carotid artery structure and stiffness

In addition to WI measures, multiple measures of carotid structure and stiffness collected during the Phase 7 clinic (2002–04) were assessed in order to investigate the relationship between other carotid phenotypes and cognitive decline. Full details of these measures can be found in the Supplementary material online, *File S1*.

### Blood pressure

Ipsilateral systolic and diastolic blood pressures (SBP and DBP, respectively) were measured in the supine position using an OMRON M5-I digital sphygmomanometer at the time of each WI assessment. Two measures of SBP and DBP were taken and their mean was used. Pulse pressure was calculated as SBP − DBP, and mean arterial pressure was calculated as DBP + (PP/3).

### Cognitive function

The cognitive test battery was introduced to the study in 1997 and has been repeated a further four times until 2016. In the current study, change in cognitive function was assessed using all four phases of data collected in clinical examinations between 2002–04 and 2015–16 (Phases 7, 9, 11, and 12). The test battery was carried out in a single-session at each time point and provides a comprehensive assessment of cognitive function covering memory, executive function, and fluency. The primary outcome of this study was a global measure of cognitive function incorporating all tests described above. More details on all cognitive measures can be found in the Supplementary material online, *File S1*.

### Covariates

Full details of all sociodemographic variables, genetic risk factors, health behaviours, and health-related covariates can be found in the Supplementary material online, *File S1*.

### Statistical analysis

Linear mixed models were used to estimate the association of carotid FCWI with change in global cognitive scores and the individual cognitive domains across the four phases of data collection between 2002–04 and 2015–16. These models use all available cognitive function data and account for the correlation between repeated measures within individuals. The intercept and slope were fitted with time as random effects for individual differences in cognitive function at baseline and rate of change of cognitive function scores over the follow-up. Associations of carotid FCWI with cognitive decline were estimated by fitting FCWI by time interaction terms in the models. Tests for interaction between sex and FCWI were all non-significant (*P* > 0.20). All analyses were therefore conducted with men and women combined, while adjusting for possible confounding effects of sex, age (linear and squared terms), ethnicity, and education by fitting these terms and their interactions with follow-up time. The models were fitted to estimate change in cognitive function over a 10-year period. Initial analyses used FCWI as a continuous measure, and then to test our hypothesis of greater effects of high FCWI, subsequent analyses examined the form of the association across levels of FCWI by categorizing the distribution into quartiles and then comparing participants with the highest 25% of FCWI with the remaining 75%.

In order to examine FCWI as a predictor of the most pronounced cognitive decline, we used the linear mixed model above, with no covariates, to estimate cognitive decline across the four waves of data collection for each participant. The 15% of participants with the greatest cognitive declines were categorized as having the binary outcome of interest. Logistic regression models were fitted and odds ratios (95% confidence intervals) calculated to estimate the association between FCWI and the fastest rate of cognitive decline. These models were initially adjusted for age and sex and a series of further models were fitted that additionally adjusted for other covariates. To assess the robustness of our findings, sensitivity analyses were conducted where threshold for the proportion of participants with the fastest cognitive decline was varied between 10 and 25%. Statistical significance was inferred at a two-tailed *P*-value <0.05.

## Results

### Participant characteristics

Sample selection is shown in *Figure 1* and full demographic and clinical characteristics are shown in *Table 1*. Of the 10 308 participants at study inception, 4105 had FCWI evaluated in 2003–05, and 3191 of these had at least two measures of cognitive function between 2002–04 and 2015–16 (four measures, *n* = 2499; three measures, *n* = 419; and two measures, *n* = 273). The final sample of 3191 individuals was predominantly white (93%) and male (75%), with a mean age of 60.7 ± 5.8 years at the measurement of FCWI.

**Table 1:** Baseline characteristics of 3191 participants in the study sample according to quartiles of carotid forward compression wave intensity

	Mean (SD) or %				*P*-value for heterogeneity
	Baseline carotid FCWI quartile				
	Quartile 1 (lowest)	Quartile 2	Quartile 3	Quartile 4 (highest)	
Carotid FCWI range (mmHg.m/s^3^)	<6075	6075–8149	8150–10 949	>10 949	
Number	781	813	811	786	
Age (years)	61.2 (5.8)	60.3 (5.6)	60.7 (5.8)	60.8 (5.9)	0.01
Female (%)	39.1	26.2	18.3	17.1	<0.001
Ethnicity					0.07
White	92.3	94.1	93.5	93.0	
Non-White	7.7	5.9	6.5	7.0	
BMI (kg/m^2^)					<0.001
Underweight (<20.0)	4.4	3.6	2.2	2.2	
Normal weight (20.0–24.9)	39.8	36.8	38.2	28.1	
Overweight (25.0–29.9)	38.9	38.2	45.5	48.4	
Obese (≥30.0)	16.9	28.1	14.1	21.4	
Carotid FCWI (mmHg.m/s^3^)	4795 (923)	7115 (582)	9401 (780)	14 949 (4563)	<0.001
Blood pressure (mmHg)^a^					
Systolic blood pressure	122.7 (12.9)	127.4 (13.8)	130.3 (13.4)	137.0 (14.2)	<0.001
Pulse pressure	44.9 (7.3)	49.5 (7.3)	52.7 (7.6)	58.4 (8.8)	<0.001
Diastolic blood pressure	77.8 (8.6)	78.0 (9.2)	77.6 (8.8)	78.6 (8.9)	0.18
Mean arterial pressure	92.8 (9.6)	94.5 (10.4)	95.2 (9.9)	98.0 (10.1)	<0.001
Education					0.09
≤Lower secondary	37.9	34.3	31.3	32.2	
Higher secondary	27.3	28.5	29.5	27.2	
≥Degree	34.8	37.2	39.2	40.6	
Employment grade					0.01
High	44.6	49.2	50.1	51.2	
Intermediate	45.8	43.9	44.8	41.6	
Low	9.6	6.9	5.2	7.3	
Smoking habit					0.02
Never	49.3	47.5	47.0	52.3	
Ex-smoker	42.3	46.0	45.9	43.3	
Current	8.5	6.5	7.2	4.5	
Alcohol consumption					0.36
No alcohol	15.5	13.0	11.8	12.5	
Moderate alcohol	64.3	65.7	68.7	66.4	
Heavy alcohol	20.2	21.3	19.5	21.1	
Moderate or vigorous physical activity (h/week)					0.67
<1	20.2	20.4	21.5	21.8	
1–6.9	62.3	63.6	61.8	64.1	
≥7	17.4	16.0	16.8	14.1	
GHQ caseness (%)	19.6	17.3	19.4	20.1	0.51
Hypertension (%)	33.0	29.6	35.0	44.8	<0.001
Diabetes (%)	5.8	4.3	6.5	10.6	<0.001
Atrial fibrillation (%)	1.9	1.0	0.1	0.9	0.004
History of CVD (%)	6.9	5.3	6.4	5.2	0.39
Physical component score	48.6 (8.9)	49.7 (8.3)	50.0 (7.6)	50.0 (7.6)	<0.001

a Blood pressure measured at the time when carotid wave intensity was measured.

BMI, body mass index; CVD, cardiovascular disease; FCWI, forward compression wave intensity; GHQ, general health questionnaire.

### Associations between forward compression wave intensity measured in mid- to late-life and changes in cognitive function

Mean [standard deviation (SD)] 10-year decline in standardized global cognitive score was 0.39 (0.18). In linear mixed models adjusting for age, age-squared, sex, ethnicity, and education; increased FCWI at baseline was independently associated with a decrease in global cognitive score during prospective follow-up [difference in 10-year change in score per 1 SD increase in FCWI = −0.02 (−0.04 to −0.00); *P* = 0.03]. Other markers of carotid structure and stiffness showed no association with any measures of cognitive function (Supplementary material online, *Table S2*).

**Figure 3:**
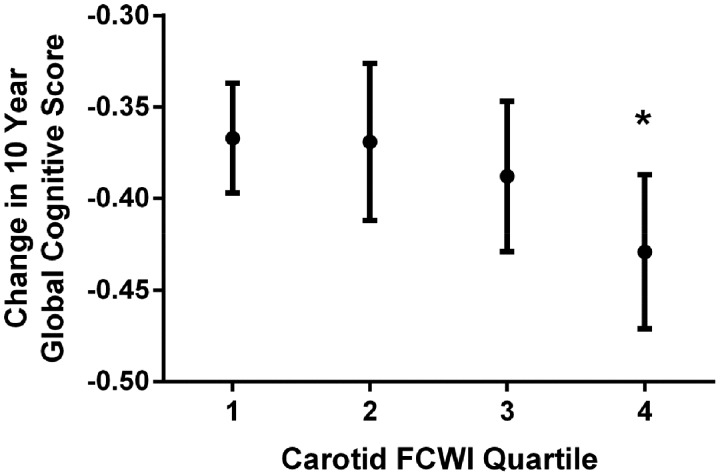
Change in 10-year global cognitive *z*-score per quartile of carotid forward compression wave intensity. FCWI, forward compression wave intensity. Means are adjusted for age, sex, ethnicity, and education and are presented for white men aged 60 years with tertiary education. *P-value = 0.04 (comparing Quartile 4 with Quartile 1).

When stratified by quartiles of FCWI, this decline was observed only in individuals with the highest FCWI at baseline [difference in 10-year change in score for Q2, Q3, and Q4 vs. Q1 = 0.01 (−0.04 to 0.06), *P* = 0.77; −0.01 (−0.06 to 0.04), *P* = 0.69; and −0.05 (−0.10 to 0.00), *P* = 0.04, respectively; *Table 2* and *Figure 3*]. As a result, individuals with the highest levels of FCWI (Q4) displayed a 13% [(−0.05/−0.38) × 100] greater decline in cognitive function [−0.05 (−0.10 to −0.01), *P* = 0.01] than the rest of the cohort combined (Q1–Q3), equivalent to an age effect of 1.9 years. Changes in executive function [AH4 −0.04 (−0.07 to −0.00); 29% greater; *P* = 0.03] and phonemic fluency [−0.09 (−0.14 to −0.03); 39% greater; *P* = 0.004] appeared to underlie this decline (*Table 2*). Adjustment for ApoE genotype had no effect on this relationship, and there was no evidence for any interaction between these two factors and cognitive outcomes.

**Table 2:** Association between carotid forward compression wave intensity and change in standardized scores per 10 years for global cognitive score and individual cognitive domains

	Change^a^ in standardized cognitive score per 10 years (95% CI), *P*-value				
Carotid FCWI	Global cognitive score	Memory	AH4	Phonemic fluency	Semantic fluency
Effect per 1 SD increase	−0.02 (−0.04, −0.00) *P* = 0.03	−0.02 (−0.05 to 0.01) *P* = 0.10	−0.01 (−0.02 to 0.01) *P* = 0.24	−0.03 (−0.05 to 0.00) *P* = 0.06	−0.01 (−0.03 to 0.02) *P* = 0.56
Quartile 1^b^	0.0 (reference)	0.0 (reference)	0.0 (reference)	0.0 (reference)	0.0 (reference)
Quartile 2	0.01 (−0.04 to 0.06) *P* = 0.77	−0.07 (−0.14 to 0.01) *P* = 0.09	0.05 (0.01 to 0.09) *P* = 0.02	0.02 (−0.05 to 0.09) *P* = 0.64	0.01 (−0.05 to 0.08) *P* = 0.75
Quartile 3	−0.01 (−0.06 to 0.04) *P* = 0.69	−0.07 (−0.14 to 0.01) *P* = 0.10	0.01 (−0.03 to 0.06) *P* = 0.44	0.02 (−0.05 to 0.09) *P* = 0.54	−0.01 (−0.08 to 0.05) *P* = 0.70
Quartile 4	−0.05 (−0.10, −0.00) *P* = 0.04	−0.07 (−0.15 to 0.01) *P* = 0.08	−0.02 (−0.06 to 0.03)*P* = 0.44	−0.07 (−0.14 to 0.00) *P* = 0.05	−0.01 (−0.08 to 0.05) *P* = 0.71
Quartiles 1–3 (lowest 75%)^c^	0.0 (reference)	0.0 (reference)	0.0 (reference)	0.0 (reference)	0.0 (reference)
Quartile 4 (highest 25%)	−0.05 (−0.09, −0.01) *P* = 0.01	−0.02 (−0.09 to 0.04) *P* = 0.45	−0.04 (−0.07, −0.00) *P* = 0.03	−0.09 (−0.14, −0.03) *P* = 0.004	−0.01 (−0.06 to 0.04) *P* = 0.67

a Changes adjusted for age, age-squared, sex, ethnicity, and education.

b Changes in 10-year cognitive score in Quartile 1 are −0.38 for global cognitive score; −0.45 for memory; −0.17 for AH4; −0.24 for phonemic fluency; and −0.27 for semantic fluency.

c Changes in 10-year cognitive score in Quartiles 1–3 are −0.38 for global cognitive score; −0.50 for memory; −0.14 for AH4; −0.23 for phonemic fluency; and −0.27 for semantic fluency.

In analysis of rapid cognitive decline as the outcome (binary variable of top 15% vs. rest), individuals with the highest FCWI (Q4) in mid- to late-life were almost 50% more likely to exhibit rapid declines in cognitive function during prospective follow-up [OR = 1.49 (1.21–1.83)], with these findings once again remaining unchanged following adjustment for all covariates [OR = 1.49 (1.17–1.88); *Table 3*]. Sensitivity analyses assessing different cut-offs for cognitive decline at 10, 15, 20, and 25% were also conducted and were broadly consistent with main findings, with full results provided in Supplementary material online, *Table S4*.

**Table 3:** Association between high baseline carotid forward compression wave intensity and accelerated cognitive decline during follow-up

		Baseline FCWI (*N* = 3191)	
		Lowest 75% (*N* = 2405)	Highest 25% (*N* = 786)
Outcome	The 15% of participants with the greatest cognitive decline over the follow-up	341 (14.2%)	155 (19.7%)
Model	Adjustments	Odds ratio (95% CI)	
M0	Unadjusted	1.0	1.49 (1.21, 1.83)
M1	M0 + age, sex	1.0	1.51 (1.21–1.88)
M2	M1 + ethnicity, education, employment grade	1.0	1.51 (1.21–1.88)
M3	M2 + smoking, alcohol consumption, physical activity	1.0	1.50 (1.20–1.87)
M4	M3 + systolic blood pressure	1.0	1.51 (1.20–1.91)
M5	M4 + GHQ caseness, hypertension, diabetes, BMI category, history of CVD, atrial fibrillation, physical component score	1.0	1.49 (1.17–1.88)

BMI, body mass index; CVD, cardiovascular disease; FCWI, forward compression wave intensity; GHQ, general health questionnaire.

## Discussion

In this longitudinal study of over 3000 individuals, we show that increased carotid FCWI in mid- to late-life is associated with greater 10-year cognitive decline. Stratification into quartiles of FCWI revealed a threshold effect for this relationship, with individuals in the highest quartile for FCWI at baseline observed to have 50% greater odds of rapid cognitive decline compared to the rest of the cohort. Importantly, this risk remained unchanged following adjustments for multiple potential confounding factors such as demographic and educational differences, genetic risk factors, hypertension and other CVD risk factors, and health-related behaviours.

Increasing evidence suggests that the disease process underlying the development of dementia, both Alzheimer’s and other forms, may begin decades before the first signs of overt cognitive impairment.^18^ Recent findings from both Whitehall II and other studies have shown that this process is likely accelerated by exposure to cardiovascular risk factors such as dyslipidaemia, diabetes, history of CVD, arrhythmias (atrial fibrillation), smoking, alcohol use, and hypertension.^10^^,^^19–22^ From mid-life onwards, the most common form of hypertension is isolated systolic hypertension—a condition attributed to a reduction in aortic compliance and characterized by an increase in SBP, a progressive decrease in DBP, and a widening of PP. These changes are a consequence of structural alterations within the major elastic arteries—particularly the aorta—and may result in an increased penetrance of pulsatile pressure and flow into the fragile microcirculation of the brain and the subsequent development of cerebral small vessel disease.^13^ Greater burden of cerebral small vessel disease, in turn, has been linked to parenchymal atrophy and the development of amyloid plaques, neuroinflammation, and neurofibrillary tangles which are commonly observed in the advanced stages of vascular dementia and Alzheimer’s disease, respectively.^14^

Previous research from the Conduit Haemodynamics of Omapatrilat International Research Study (CHOIR) has suggested that increased forward-travelling compression waves due to elevated aortic characteristic impedance is the predominant factor underlying the increases in PP commonly observed from mid-life, with this phenomenon involving a combination of aortic remodelling and progressive increases in aortic stiffness that commonly occur in middle-age.^23^ While several smaller cross-sectional and some prospective studies have shown relationships between surrogate markers of arterial stiffness/pulsatility and cognitive performance^2–8^; no previous study to date has directly investigated the relationship between the intensity of these compression waves approaching the brain and cognitive decline. In order to assess directly this relationship, we used carotid wave intensity analysis—a non-invasive technique that allows the forwards- and backwards-travelling waves within the major arteries supplying the brain to be separated—to quantify the peak rate of energy transfer transmitted towards the fragile cerebral microcirculation with each beat of the heart.^17^

This novel technique has enabled us to conduct a large-scale study of the relationship between FCWI and cognitive outcomes in an extensively-phenotyped longitudinal cohort. We have shown that individuals with higher carotid FCWI in mid- to late-life are at greater risk of accelerated cognitive decline during 11–14-year follow-up. Importantly, although higher FCWI was associated with a number of other mid-life CVD risk factors also associated with dementia (e.g. increased body mass index, systolic hypertension, diabetes),^24^ adjustment for these and other potential confounding factors had little impact on the relationship between FCWI and decline in cognitive function. Furthermore, this association did not appear to result from structural and functional changes within the carotid artery itself, as no relationship was observed between any carotid phenotype and cognitive decline. These findings support previous work suggesting that an increase in forward wave amplitude within the carotid arteries is likely attributable to altered ventricular dynamics and/or disproportionate changes in aortic characteristic impedance or compliance,^23^^,^^25^^,^^26^ as opposed to increases in carotid stiffness *per se.* Together, these findings suggest that exposure to increased wave intensity in mid- to late-life may contribute to the observed association between arterial stiffness in mid-life and risk of dementia in the following decades, and that this relationship cannot be detected using common carotid phenotypes.

There are a number of strengths in our study to support this hypothesis. Firstly, the Whitehall II cohort represents the largest and most extensively characterized population to date containing both carotid wave intensity measures collected during mid- to late-life and long-term serial measures of cognitive function assessed throughout old age. Although the observational nature of this cohort meant it was not possible to infer causal associations between exposures and outcomes, it provided a unique opportunity to investigate—for the first time—the likely haemodynamic mechanisms linking increased blood pressure to cerebral small vessel disease. These data may provide insight into the potential mechanistic process underlying recent results from the SPRINT-MIND trial, in which the risk for mild cognitive impairment was reduced following intensive blood pressure treatment.^27^ Secondly, the extensive phenotyping of individuals collected over four decades in the Whitehall II study, alongside high retention rates, allowed these relationships to be tested independently of a wide-range of genetic, social, and health-related risk factors which have previously been linked to future incidence of dementia in this cohort, and which may have therefore confounded results.^10^^,^^20–22^^,^^28^ Thirdly, although the limited numbers of participants with WI measures (∼50% of Phase 7 population) meant that our study was not adequately powered to investigate dementia itself as a primary outcome, an accelerated decline in the global cognitive score employed in this study has previously been shown to occur in the 8–10 years prior to a diagnosis of dementia.^10^ This score therefore provides an earlier-detectable and clinically relevant measure of disease progression that may be targeted in interventional studies long before clinical diagnosis of dementia. In addition, the emergence of phonemic fluency as the predominant factor underlying these changes supports previous findings from this cohort linking arterial pulsatility to cognitive decline, with prospective changes in this cognitive domain found to be most closely related to high levels of SBP in women, and to be the only domain linked to SBP in men, during early middle-age (mean age 44 ± 6 years).^28^ There are a number of potential limitations to the current study. Firstly, given the predominantly white (93%) and male (75%) demographic of the Whitehall II cohort, future studies reproducing these findings in female or more ethnically diverse cohorts are required in order to establish whether this measure may potentially impact future clinical practice. Secondly, significant cohort differences exist between participants who responded to the invitation to attend the WI clinic and those who did not. However, as those in attendance tended to be healthier overall, it is not unreasonable to predict that the changes observed here would be likely to be equivalent or even greater in the cohort as a whole. Thirdly, brain imaging was not available in all members of this cohort, and it was therefore not possible to relate cerebral structural and functional changes to cognitive decline. However, future work in a sub-sample of ∼600 participants from this cohort who have recently undergone detailed MRI scans and are now being recalled for repeat testing are expected to shed future light on these relationships.^29^ Finally, the use of brachial blood pressure as a surrogate for carotid blood pressure may have introduced a source of bias when calculating wave intensity. However, these measures have previously been shown to be equivalent in elderly populations, making this an unlikely limitation.^16^

Our findings provide support for a link between cardiovascular health in mid- to late-life and risk of dementia in later life, and therefore, have important clinical implications. Given the well-publicized failure of multiple Phase III dementia outcome trials carried out in the later stages of the disease,^30^^,^^31^ our findings highlight the importance of early strategies to slow or prevent vascular disease and cognitive decline.

In conclusion, we provide the first evidence of a link between the proposed mechanism hypothesized to cause cerebral small vessel damage (i.e. increased wave intensity transmitted into the cerebral circulation) and an accelerated decline in cognitive function. In addition, as FCWI remained an independent predictor of future cognitive decline even after adjustment for an extensive range of potential confounding factors, interventions to lower carotid FCWI may hold promise for delaying or preventing the onset of cognitive decline.

## Supplementary Material

ehz189_Supplementary_Data

## Notes

See page 2310 for the editorial comment on this article (doi: 10.1093/eurheartj/ehz307)
