# Supplementary material for: Carotid artery wave intensity in mid- to late-life predicts cognitive decline: the Whitehall II study
Source: Eur Heart J. 2019 Apr 8;40(28):2300–9. doi: 10.1093/eurheartj/ehz189 (PMC6642727; doi:10.1093/eurheartj/ehz189)
Supplement: ehz189_Supplementary_Data [file ehz189_supplementary_data.docx]

**SUPPLEMENTARY FILE**

**Chiesa et al.**

**Carotid artery wave intensity in mid- to late-life predicts cognitive decline: The Whitehall II study**

**SUPPLEMENTARY METHODS**

**Carotid Structure and Stiffness**

Measurements were taken in a quiet, temperature-controlled room (22–26°C) using an Aloka 5500 high-resolution ultrasound system with 7.5 MHz transducer. Participants were examined in a supine position with the head positioned at a ~45° angle. Longitudinal images of the common carotid artery, triggered on the R-wave of the ECG, were recorded in DICOM format as a cine loop on the hard drive of the ultrasound machine for later analysis.

***Carotid Intima-Media Thickness (cIMT):*** Intima-media thickness (IMT) was measured in the right and left common carotid arteries, and the mean was used for analysis. The common carotid IMT was measured at its thickest part 1 cm proximal to the bifurcation. A measurement was taken between the leading edge of the intima and the media adventitia on three separate images on each side using electronic callipers, and the mean of the six measures was used for analysis.

***Carotid Stiffness:*** The following measures of carotid stiffness were also calculated in order to assess the relationship between established markers of vessel stiffness and changes in cognitive function:

Carotid Distensibility = (D_max-D_min) / D_min

Carotid Compliance = (D_max-D_min) / (P_max-P_min)

Beta Stiffness = ln (P_max/P_min) / [(D_max-D_min)/D_min)]

Elastic modulus = (P_max-P_min) / [(D_max-D_min)/D_min]

where D_max = maximum vessel diameter during systole, D_min = minimum vessel diameter during diastole, P_Max = systolic blood pressure measured during scan, and P_min = diastolic blood pressure measured during scan. Measures were made in both left and right carotid arteries, and the mean was used for analysis.

**Cognitive Test Battery and Global Score**

*Memory*: assessed using a 20 word free-recall test. Participants were presented with a list of one or two syllable words at two second intervals and were then asked to recall in writing as many of the words as possible in any order within two minutes.

*Executive function*: assessed with the Alice Heim 4-I test, which is composed of a series of 65 verbal and mathematical reasoning items of increasing difficulty. It tests inductive reasoning, measuring the ability to identify patterns and infer principles and rules. Participants had 10 minutes to do this section.

*Fluency*: assessed using measures of phonemic and semantic fluency. Participants were asked to recall in writing as many words beginning with “s” (phonemic fluency) and as many animal names (semantic fluency) as they could. One minute was allowed for each test.

*Global cognitive score:* achieved by first using the distribution of the cognitive data at baseline (2002-2004) to standardise the raw scores for each domain to z scores (mean=0; standard deviation=1). These scores were then summed and re-standardised to yield the global score, an approach that minimises measurement error inherent in individual tests^1^. The cognitive data from the subsequent three phases were then standardised using the means and standard deviations from the 2002-2004 data to maintain comparability across the phases.

**Covariates**

Sociodemographic variables included age, sex, ethnicity (white, south Asian, black, other), socioeconomic status (three categories: high, intermediate, and low employment grade, representing income and status at work) and education (three categories: less than lower secondary school (up to age 16), higher secondary school (up to age 18), and university degree or higher).

ApoE genotype was determined as previously described^2^. Genotype was read blindly by two independent observers, and any discrepancies were resolved by repeating PCR analysis.

Health behaviours were assessed by questionnaire and included smoking (current, ex-, and never smokers), alcohol consumption (number of alcoholic drinks consumed in previous seven days, converted to units of alcohol per week and categorised as “no/occasional consumption,” “moderate consumption” (1-14 units/week in women, 1-21 units/week in men), and “heavy consumption” (>14 units in women, >21 units in men), and physical activity (<1hr/week, 1 – 6.9hrs/week, and ≥7hrs/week).

Health related covariates included symptoms of anxiety and depression (30 item General Health Questionnaire), hypertension (SBP/DBP ≥140/90mmHg or use of antihypertensive drugs), prevalent diabetes mellitus (determined by fasting glucose ≥7.0mmol/L or 2-h postload glucose ≥11.1mmol/L, reported diabetes diagnosed by a doctor, or use of diabetes drugs), body mass index assessed at the clinical examination (categorised as <20, 20-24.9, 25-29.9, and ≥30kg/m^2^), prevalent cardiovascular diseases (including coronary heart disease and stroke identified using linkage to national hospital records), and the physical component score of the SF-36 (self-rated physical functioning).

**SUPPLEMENTARY TABLES**

**Table S1: Comparison of characteristics of 6483 participants who attended Phase 7 screening clinic according to whether they are in the study sample**

|  | Study sample | | p-value |
| --- | --- | --- | --- |
|  | Excluded | Included |  |
|  | [Mean (SD) or %] | [Mean (SD) or %] |  |
|  |  |  |  |
| Number | 3292 | 3191 |  |
|  |  |  |  |
| Age at Phase 7 clinic, y | 61.5 (6.1) | 60.7 (5.8) | <0.001 |
| Female, % | 33.1 | 25.1 | <0.001 |
| Ethnicity |  |  | <0.001 |
| - *White* | 90.3 | 93.2 |  |
| - *Non-White* | 9.7 | 6.8 |  |
| BMI (kg/m^2^) |  |  | <0.001 |
| - *Underweight (<20.0)* | 2.7 | 3.1 |  |
| - *Normal weight (20.0 - 24.9)* | 30.9 | 35.8 |  |
| - *Overweight (25.0 – 29.9)* | 45.1 | 44.5 |  |
| - *Obese (≥ 30.0)* | 21.3 | 16.7 |  |
| Blood Pressure (mmHg)^a^ |  |  |  |
| - *Systolic Blood Pressure* | 130.0 (17.9) | 126.4 (15.7) | <0.001 |
| - *Pulse Pressure* | 54.8 (12.4) | 52.7 (10.4) | <0.001 |
| - *Diastolic Blood Pressure* | 75.2 (10.7) | 73.7 (10.2) | <0.001 |
| - *Mean Arterial Pressure* | 93.5 (12.2) | 91.3 (11.3) | <0.001 |
|  |  |  |  |
| Education |  |  | 0.004 |
| - *≤ Lower secondary* | 37.9 | 33.9 |  |
| - *Higher secondary* | 27.0 | 28.1 |  |
| - *≥ Degree* | 35.1 | 38.0 |  |
| Employment grade |  |  | <0.001 |
| - *High* | 42.2 | 48.8 |  |
| - *Intermediate* | 43.2 | 44.0 |  |
| - *Low* | 14.7 | 7.2 |  |
| Smoking habit |  |  | <0.001 |
| - *Never* | 47.7 | 49.0 |  |
| - *Ex-smoker* | 42.7 | 44.4 |  |
| - *Current* | 9.6 | 6.6 |  |
| Alcohol consumption |  |  | <0.001 |
| - *No alcohol* | 20.6 | 13.2 |  |
| - *Moderate alcohol* | 60.6 | 66.3 |  |
| - *Heavy alcohol* | 18.8 | 20.5 |  |
| Moderate or vigorous physical activity |  |  | <0.001 |
| - *< 1 hr/wk* | 26.6 | 21.0 |  |
| - *1 - 6.9 hrs/wk* | 58.8 | 63.0 |  |
| - *≥ 7 hrs/wk* | 14.6 | 16.1 |  |
|  |  |  |  |
| GHQ caseness, % | 21.6 | 19.1 | 0.01 |
| Hypertension, % | 44.3 | 35.6 | <0.001 |
| Diabetes, % | 10.1 | 6.8 | <0.001 |
| Atrial fibrillation, % | 1.5 | 1.0 | 0.05 |
| History of CVD, % | 6.4 | 6.0 | 0.42 |
| Physical component score | 47.9 (9.7) | 49.6 (8.1) | <0.001 |

^a^ Blood pressure measures from Whitehall Phase 7 screening clinic (2002-2004)

Abbreviations: BMI, body mass index; FCWI, forward compression wave intensity; GHQ, general health questionnaire; CVD, cardiovascular disease.

**Table S2: Association between carotid structure and stiffness and cognitive decline**

|  | Change in standardised cognitive function per 10 years associated with 1SD change in carotid phenotype (95% CI), p-value | | | | |
| --- | --- | --- | --- | --- | --- |
|  | Global cognitive score | Memory | AH4 | Phonemic fluency | Semantic fluency |
| Intima-media thickness | 0.007 (-0.011, 0.025)  p=0.43 | -0.014 (-0.041, 0.013)  p=0.30 | 0.002 (-0.013, 0.017)  p=0.79 | 0.026 (0.001, 0.051)  p=0.04 | 0.011 (-0.012, 0.033)  p=0.36 |
| Mean carotid distensibility | 0.001 (-0.016, 0.019)  p=0.88 | 0.006 (-0.021, 0.034)  p=0.64 | -0.003 (-0.018, 0.012)  p=0.70 | 0.005 (-0.021, 0.030)  p=0.72 | -0.008 (-0.031, 0.015)  p=0.52 |
| Mean carotid compliance | 0.009 (-0.025, 0.010)  p=0.40 | -0.004 (-0.030, 0.022)  p=0.75 | -0.010 (-0.025, 0.005)  p=0.18 | 0.001 (-0.024, 0.025)  p=0.96 | -0.013 (-0.035, 0.009)  p=0.26 |
| β stiffness Index | 0.004 (-0.014, 0.023)  p=0.65 | -0.003 (-0.031, 0.025)  p=0.83 | 0.007 (-0.008, 0.023)  p=0.35 | 0.004 (-0.022, 0.030)  p=0.76 | 0.007 (-0.016, 0.030)  p=0.53 |
| Elastic Modulus | -0.001 (-0.020, 0.017)  p=0.87 | -0.008 (-0.036, 0.020)  p=0.58 | 0.001 (-0.014, 0.017)  p=0.88 | -0.002 (-0.028, 0.024)  p=0.88 | 0.006 (-0.017, 0.030)  p=0.59 |

Cognitive changes are adjusted for age, age squared, sex, ethnicity and education

**Table S3: Baseline characteristics of 3191 participants comparing those in highest quartile of carotid wave intensity with those in the lowest three quartiles.**

|  | [Mean (SD) or %] | | p-value |
| --- | --- | --- | --- |
|  | Baseline carotid FCWI | |  |
|  | Lowest 75%  (Q1-Q3) | Highest 25%  (Q4) |  |
|  |  |  |  |
| Number | 2405 | 786 |  |
|  |  |  |  |
| Age, y | 60.7 (5.7) | 60.8 (5.9) | 0.59 |
| Female, % | 27.8 | 17.1 | <0.001 |
|  |  |  |  |
| Ethnicity |  |  | 0.77 |
| - *White* | 93.3 | 93.0 |  |
| - *Non-White* | 6.7 | 7.0 |  |
| BMI (kg/m^2^) |  |  | <0.001 |
| - *Underweight (<20.0)* | 3.4 | 2.2 |  |
| - *Normal weight (20.0 - 24.9)* | 38.3 | 28.1 |  |
| - *Overweight (25.0 – 29.9)* | 43.2 | 48.4 |  |
| - *Obese (≥ 30.0)* | 15.1 | 21.4 |  |
|  |  |  |  |
| Carotid FCWI (mmHg.m/s^3^) | 7133 (2027) | 14949 (4563) | <0.001 |
| Blood Pressure (mmHg)^a^ |  |  |  |
| - *Systolic Blood Pressure* | 126.9 (13.7) | 137.0 (14.2) | <0.001 |
| - *Pulse Pressure* | 49.1 (8.1) | 58.4 (8.8) | <0.001 |
| - *Diastolic Blood Pressure* | 77.8 (8.8) | 78.6 (8.9) | 0.04 |
| - *Mean Arterial Pressure* | 94.2 (10.0) | 98.0 (10.1) | <0.001 |
|  |  |  |  |
| Education |  |  | 0.21 |
| - *≤ Lower secondary* | 34.5 | 32.2 |  |
| - *Higher secondary* | 28.4 | 27.2 |  |
| - *≥ Degree* | 37.1 | 40.6 |  |
| Employment grade |  |  | 0.27 |
| - *High* | 48.0 | 51.2 |  |
| - *Intermediate* | 44.8 | 41.6 |  |
| - *Low* | 7.2 | 7.3 |  |
| Smoking habit |  |  | 0.006 |
| - *Never* | 47.9 | 52.3 |  |
| - *Ex-smoker* | 44.7 | 43.3 |  |
| - *Current* | 7.4 | 4.5 |  |
| Alcohol consumption |  |  | 0.74 |
| - *No alcohol* | 13.4 | 12.5 |  |
| - *Moderate alcohol* | 66.2 | 66.4 |  |
| - *Heavy alcohol* | 20.3 | 21.1 |  |
| Moderate or vigorous physical activity |  |  | 0.22 |
| - *< 1 hr/wk* | 20.7 | 21.8 |  |
| - *1 - 6.9 hrs/wk* | 62.6 | 64.1 |  |
| - *≥ 7 hrs/wk* | 16.7 | 14.1 |  |
|  |  |  |  |
| GHQ caseness, % | 18.8 | 20.1 | 0.40 |
| Hypertension, % | 32.6 | 44.8 | <0.001 |
| Diabetes, % | 5.5 | 10.6 | <0.001 |
| Atrial fibrillation, % | 1.0 | 0.9 | 0.79 |
| History of CVD, % | 6.2 | 5.2 | 0.31 |
| Physical component score | 49.4 (8.3) | 50.0 (7.6) | 0.08 |

^a^ Blood pressure measured at the time when carotid wave intensity was measured

Abbreviations: BMI, body mass index; FCWI, forward compression wave intensity; GHQ, general health questionnaire; CVD, cardiovascular disease.

**Table S4. Sensitivity analyses of the association between wave intensity and greatest cognitive decline using highest 10, 15, 20 and 25% of cognitive decline**

|  |  | Baseline carotid FCWI (N=3191) | |  | Odds ratio (95% CI) | | |
| --- | --- | --- | --- | --- | --- | --- | --- |
| Outcome: |  | Lowest 75% (N=2405) | Highest 25% (N=786) |  |  |  |  |
| Top percentiles of greatest cognitive decline |  | N (%) in top percentile | N (%) in top percentile |  | Unadjusted | Model 1 | Model 2 |
|  |  |  |  |  |  |  |  |
| Top 10% |  | 231 (9.6%) | 100 (12.7%) |  | 1.37 (1.07, 1.76) | 1.37 (1.05, 1.77) | 1.31 (0.99, 1.73) |
| Top 15% |  | 341 (14.2%) | 155 (19.7%) |  | 1.49 (1.21, 1.83) | 1.51 (1.21, 1.88) | 1.49 (1.17, 1.88) |
| Top 20% |  | 473 (19.7%) | 184 (23.4%) |  | 1.25 (1.03, 1.52) | 1.25 (1.02, 1.54) | 1.23 (0.99, 1.53) |
| Top 25% |  | 599 (24.9%) | 212 (27.0%) |  | 1.11 (0.93, 1.34) | 1.11 (0.91, 1.34) | 1.09 (0.88, 1.33) |
|  |  |  |  |  |  |  |  |

Model 1: adjusted for age, sex, ethnicity, education, employment grade

Model 2: adjusted as for Model 1 + SBP, GHQ caseness, hypertension, diabetes, BMI category, history of CVD, atrial fibrillation, physical component score

Abbreviations: BMI, body mass index; FCWI, forward compression wave intensity; GHQ, general health questionnaire; CVD, cardiovascular disease.

**Supplementary References**

1. Wilson RS, Leurgans SE, Boyle PA, Schneider JA, Bennett DA. Neurodegenerative basis of age-related cognitive decline. *Neurology* 2010;**75**:1070–1078.

2. Zhao JH, Brunner EJ, Kumari M, Singh-Manoux A, Hawe E, Talmud PJ, Marmot MG, Humphries SE. APOE polymorphism, socioeconomic status and cognitive function in mid-life--the Whitehall II longitudinal study. *Soc Psychiatry Psychiatr Epidemiol* 2005;**40**:557–563.
